# Safety and Tolerability of a Wearable, Vibrotactile Stimulation Device for Parkinson’s Disease

**DOI:** 10.3389/fnhum.2021.712621

**Published:** 2021-11-18

**Authors:** Laura Tabacof, Stephen Braren, Taylor Patterson, Adam Fry, David Putrino

**Affiliations:** ^1^Department of Rehabilitation and Human Performance, Icahn School of Medicine at Mount Sinai, New York, NY, United States; ^2^Department of Applied Psychology, New York University, New York, NY, United States

**Keywords:** Parkinson’s disease, resting tremor, wearable technologies, vibration, UPDRS, vibrotactile, Parkinson tremor, wearables acceptance

## Abstract

**Background:** Resting tremor is a cardinal symptom of Parkinson’s disease (PD) that contributes to the physical, emotional, and economic burden of the disease.

**Objective:** The goal of this study was to investigate the safety, tolerability, and preliminary effectiveness of a novel wearable vibrotactile stimulation device on resting tremor in individuals with PD.

**Methods:** Using a randomized cross-over design, subjects received two different vibrotactile stimulation paradigms (high amplitude patterned and low amplitude continuous) on two separate laboratory visits. On each visit, resting tremor was video recorded for 10 min at baseline and while the vibrotactile stimulation was applied. Tremor severity was scored by a blinded clinician.

**Results:** Both vibration paradigms were well safe and well tolerated and resulted in a reduction in resting tremor severity with a moderate effect size (*n* = 44, *p* < 0.001, *r* = 0.37–0.54). There was no significant difference between the two vibration paradigms (*p* = 0.14).

**Conclusion:** Short durations of vibrotactile stimulation delivered *via* wearable devices were safe and well tolerated and may attenuate resting tremor severity in individuals with PD. The sample size as well as the potential preliminary effectiveness revealed by two arms of the study could not eliminate the potential for a placebo effect.

## Introduction

Resting tremor is a highly prevalent and burdensome symptom of Parkinson’s disease (PD) (Hughes et al., 1993; Louis et al., 1997; Kowal et al., 2013). With no available cure for PD, current therapies target the symptoms of the disease. Responses of resting tremor to pharmaceutical intervention vary widely between individuals (Kalia and Lang, 2015; Pasquini et al., 2018) and variations in tremor intensity accompany medication “off” periods that occur even with extended release formulations (Ramirez-Zamora and Molho, 2014). Surgical interventions may provide more pronounced and consistent alleviation of resting tremor (Deuschl et al., 2006), but have limited clinical indications (Morgante et al., 2007; Kestenbaum et al., 2015). Therefore, auxiliary therapies for resting tremor remain highly desirable. Whole body vibration such as vibrating chairs and platforms has been investigated as a potential means to reduce resting tremor, however, results have been inconsistent (Haas et al., 2006; King et al., 2009; Kapur et al., 2012; Gaßner et al., 2014). Regardless of efficacy, such interventions do not represent a practical solution for many individuals as they are immobile, expensive and not highly customizable. If effective at lessening resting tremor, wearable vibrotactile stimulation devices may represent an attractive solution to PD patients. The aim of this pilot study was to evaluate the safety and tolerability of vibrotactile stimulation delivered *via* wearable devices on Parkinsonian resting tremor. We also aimed to collect preliminary effectiveness data on each study arm.

## Materials and Methods

### Subjects

Participants with a diagnosis of PD and resting tremor in one or both hands were enrolled in the study. All subjects provided written informed consent. The study was approved by the local Program for Protection of Human Subjects (IRB 17-00555). All study procedures took place at the Abilities Research Center at Mount Sinai Hospital between July 2017 and January 2018. Individuals with moderate to severe cognitive impairment, pre-existing essential tremor, deep-brain stimulation implant, or sensory impairments that would make their response to sensory stimulation unpredictable were excluded from the trial.

### Study Design

This feasibility study was a randomized cross-over clinical trial. Each individual was assessed on two different occasions, with a 1–14-day interval between visits. Baseline assessments involved a 10-min video recording of baseline resting tremor. The wearable vibrotactile stimulation devices were then placed over the subject’s wrists and ankles and another 10-min video recording was collected while vibrotactile stimulation was delivered. During recordings, subjects were seated with their knees and feet together, with forearms positioned on the armrests of the chair so that their hands hung unobstructed from their wrists. Subjects were instructed not to alter their medication schedule but significant effort was made to ensure that both study sessions occurred at the same time of day, under the same medication parameters for all participants. Both visits were scheduled at a similar time of day when their tremor was thought likely to be present.

### Vibrotactile Stimulation

**FIGURE 1:**
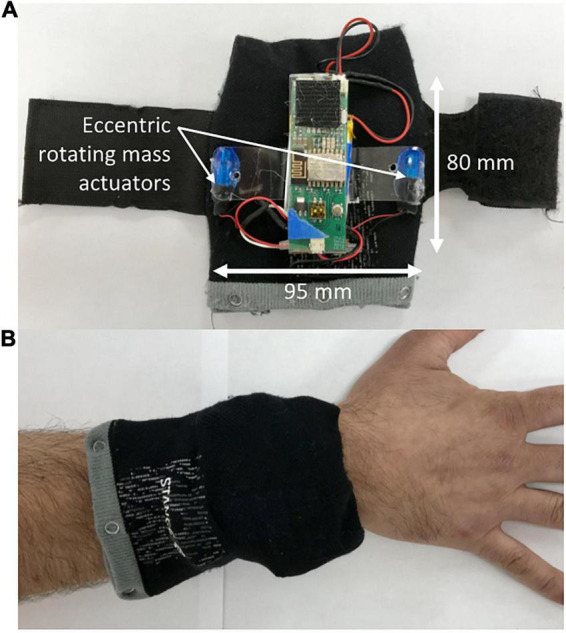
The wearable vibrotactile stimulation device. Each vibration unit powered two eccentric rotating mass actuators from which the vibrotactile stimulation was delivered **(A)**. The vibration units were housed in cloth pouches that were attached to the subject’s wrists and ankles using a Velcro strap **(B)**.

**FIGURE 2:**
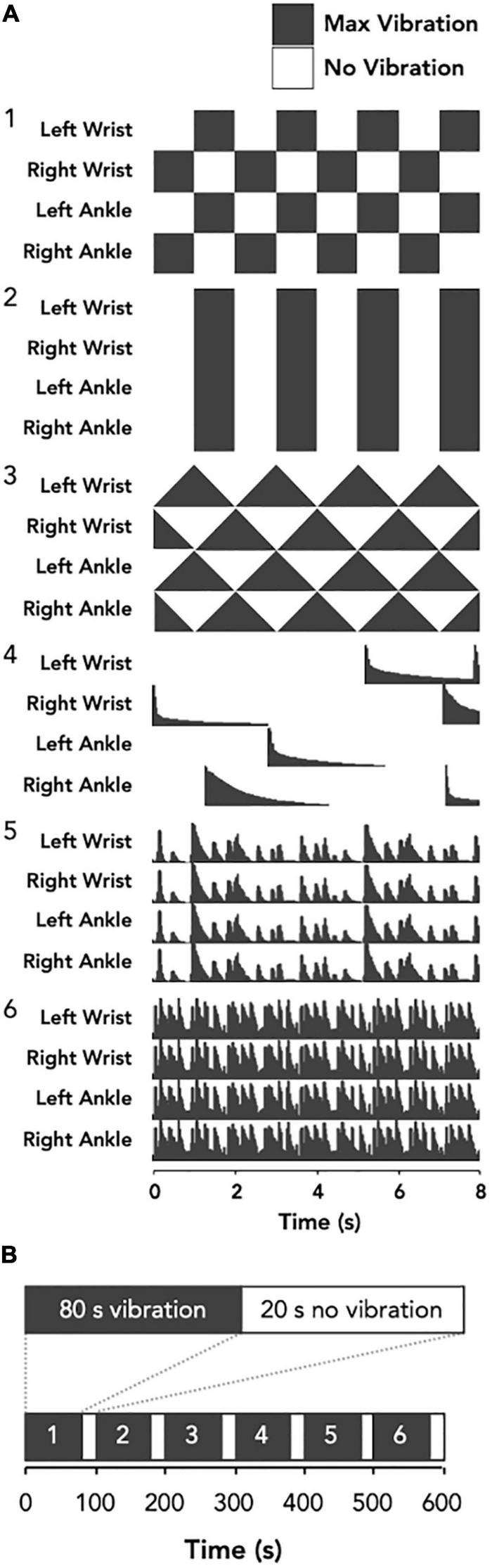
Schematic representation of the vibrotactile stimulation protocol. The vibration patterns for the HA-P vibration trial **(A)** were three crafted waveforms; left-right oscillating (1), on-off oscillating (2), and sawtooth (3), and three audio extracted waveforms; random chimes (4), slow ternary monophonic music track (5), and fast electronic music track (6). Conversely, for the LA-C vibration trial the stimulus was constant (not shown). Each bout of vibration was delivered for 80 s with 20 s off-periods separating each bout **(B)**.

The vibrotactile stimulation was applied to both wrists and ankles using four custom-built wearable devices to promote an optimal full body vibrotactile stimuli. Each device involved a vibration unit with two eccentric rotating mass actuators approximately 75 mm apart (Figure 1A), which was housed in a cloth pouch that was fastened to the limb using a Velcro strap (Figure 1B). On one visit, the devices provided six distinct vibration patterns to evaluate the overall tolerability of strong, noticeable vibrotactile stimulation paradigms (Figure 2A). The frequency of vibrations during these patterns ranged from 40 to 200 Hz. Each pattern was 80 s in duration, with 20 s separating each pattern; making a total of 10 min, and participants were given the opportunity to provide feedback about each pattern of vibrotactile stimulation (Figure 2B). During the other visit, the devices provided a continuous vibration at approximately 48 Hz to evaluate the overall tolerability of a weak, barely noticeable vibrotactile stimulation paradigm. This vibration was also applied for six 80 s blocks, with a brief pulse of vibration marking the start and end of each block, and 20 s separating each block. These two vibration paradigms are hereon referred to as high amplitude patterned (HA-P) vibration and low amplitude continuous (LA-C) vibration, respectively. Vibration intensity (full, strong, medium, or weak) was set up initially at full and adjusted according to subject’s tolerance throughout each session.

### Quantification of Tremor

Subjects were video recorded (30 Hz) using the Microsoft Kinect 2 throughout both visits. Resting tremor severity was scored according to item 20 of the Unified Parkinson’s Disease Rating Scale (UPDRS) by a clinician who was blinded to vibration status. Resting tremor severity was scored on a minute-by-minute basis throughout the four 10-min resting tremor assessments; both the baseline and vibration periods of the HA-P vibration and LA-C vibration trials.

### Statistical Analyses

We used multilevel modeling to test for within- and between-subject differences in tremor severity while accounting for the within-subject non-independence of the repeated measures. Baseline tremor scores were similar between visits, and were therefore averaged to simplify these models. Gender, age, time since diagnosis, whether the participant was currently taking Parkinsonian medication, and time since last medications dose showed no significance as covariates and were therefore removed from the model. Final analyses used three-level models with tremor severity scores at level 1, experimental condition (averaged baselines, HA-P vibration, and LA-C vibration) at level 2, and subjects at level 3. Effect sizes were computed using the standardized regression coefficients.

## Results

### Subjects

Fifty-two subjects were enrolled in the study. One subject dropped out after the first visit due to inability to tolerate the seated position, and another opted out for personal reasons not given. Six subjects did not exhibit a resting tremor in both the baseline and vibration recordings in one or both of the laboratory visits and were subsequently excluded from the analysis. Thus, data analysis was performed on the remaining 44 subjects (33/11 males/females; age: 67 ± 10 years; time since diagnosis: 6 ± 4 years). All resting tremor severity scores ranged between 0 and 3. Most participants (93%) were undergoing pharmacological treatment for Parkinsonian symptoms at the time of the study, including levodopa, dopamine agonists, and antidepressants. Time between study session and last medication dose was 4.9 ± 4.0 and 5.4 ± 4.9 h for the LA-C and HA-P vibration trials, respectively.

### Safety and Tolerance

All subjects tolerated the vibrotactile stimulation well, with no reported adverse events. Five (11%) requested decrease in vibration intensity. No subjects reported discomfort in response to the stimulation or requested early termination of the vibration. Comments and setting preferences are detailed in Table 1.

**TABLE 1:** Vibration paradigm, intensity, and comments during study visits.

	Visit 1				Visit 2			
		
Patient ID	Vibration paradigm	Wrist intensity level	Ankle intensity level	Comments	Vibration paradigm	Wrist intensity level	Ankle intensity level	Comments
1	HA-P	Full	Full		LA-C	–	–	
2	LA-C	–	–		HA-P	Full	Full	
3	HA-P	Full	Full		LA-C	–	–	
4	LA-C	–	–		HA-P	Medium	Full	
5	LA-C				HA-P	Full	Full	Protocol #3 “It will put me to sleep,” “Sounds like a car motor”; #5 “Normally when I listen to music the tremors are better”
6	HA-P	Full	Full		LA-C	–	–	
7	HA-P	Full	Full		LA-C	–	–	
8	LA-C	–	–		LA-C	–	–	
9	HA-P	Full	Full		LA-C	–	–	
10	HA-P	Full*	Full*		–	–	–	
11	LA-C	–	–		LA-C	–	–	
12	HA-P	Medium*	Full		HA-P	Full	Full	
13	LA-C	–	–		LA-C	–	–	
14	LA-C	–	–		HA-P	Full	Full	
15	HA-P	Full*	Full*	Protocol #1:“Feels like arm is being massaged” #2: “There’s a pleasant sensation through the arm” #3: “Pleasant feeling”; “I feel like I can open my hand easier” #4 “Feels less effective” #5: “Better than 4” # 6: “More relief” “I like the beat better” “Feels some relief after going through the whole protocol”	LA-C	–	–	“My arm does feel better with device on”
16	HA-P	Full*	Full*		LA-C	–	–	
17	HA-P	Full	Full		LA-C	–	–	
18	HA-P	Full	Full		LA-C	–	–	
19	LA-C	–	–	“Staying stationary in the same position is uncomfortable”	HA-P	Full	Full	
20	HA-P	Medium	Full		LA-C	–	–	
21	HA-P	Full	Full		LA-C	–	–	
22	HA-P	Full	Full		LA-C	–	–	
23	HA-P	Full	Full		LA-C	–	–	
24	LA-C	–	–		HA-P	Full	Full	
25	LA-C	–	–		HA-P	Full	Full	
26	LA-C	–	–		HA-P	Full	Full	
27	HA-P	Full	Full		LA-C	–	–	
28	LA-C	–	–		HA-P	Full	Full	
29	LA-C	–	–		HA-P	Full	Full	
30	LA-C	–	–	“With jolt stops tremors for 1–2 s”	HA-P	Full	Full	
31	LA-C	–	–		HA-P	Full	Full	
32	HA-P	Full*	Full		LA-C	–	–	Protocol #6: “The noise tended to take away from the shaking; it was a slight distraction” “Didn’t seem to be doing much”
33	HA-P	Full	Full		LA-C	–	–	
34	LA-C	–	–		HA-P	strong	Full	
35	HA-P	Full	Full	“It is disconcerting to draw spirals with the device on”	LA-C	–	–	
36	LA-C	–	–		HA-P	Full	Full	
37	LA-C	–	–		HA-P	Full	Full	
38	HA-P	Full	Full		LA-C	–	–	
39	HA-P	Full	Full		LA-C	–	–	
40	LA-C	–	–	“It feels like the vibration is stronger in the right wrist than in the left wrist”; “I got used to the vibrations at the end”	HA-P	Full	Full	
41	LA-C	–	–		HA-P	Full	Full	
42	HA-P	Full	Full	“The sound and the rough form factor is too much for the whole day”	LA-C	–	–	
43	HA-P	Full	Full		LA-C	–	–	
44	LA-C	–	–		LA-C	–	–	

*HA-P, high amplitude patterned vibration; LA-P, low amplitude continuous vibration.*

*Requested decrease in intensity.

### Effect of Vibrotactile Stimulation

**FIGURE 3:**
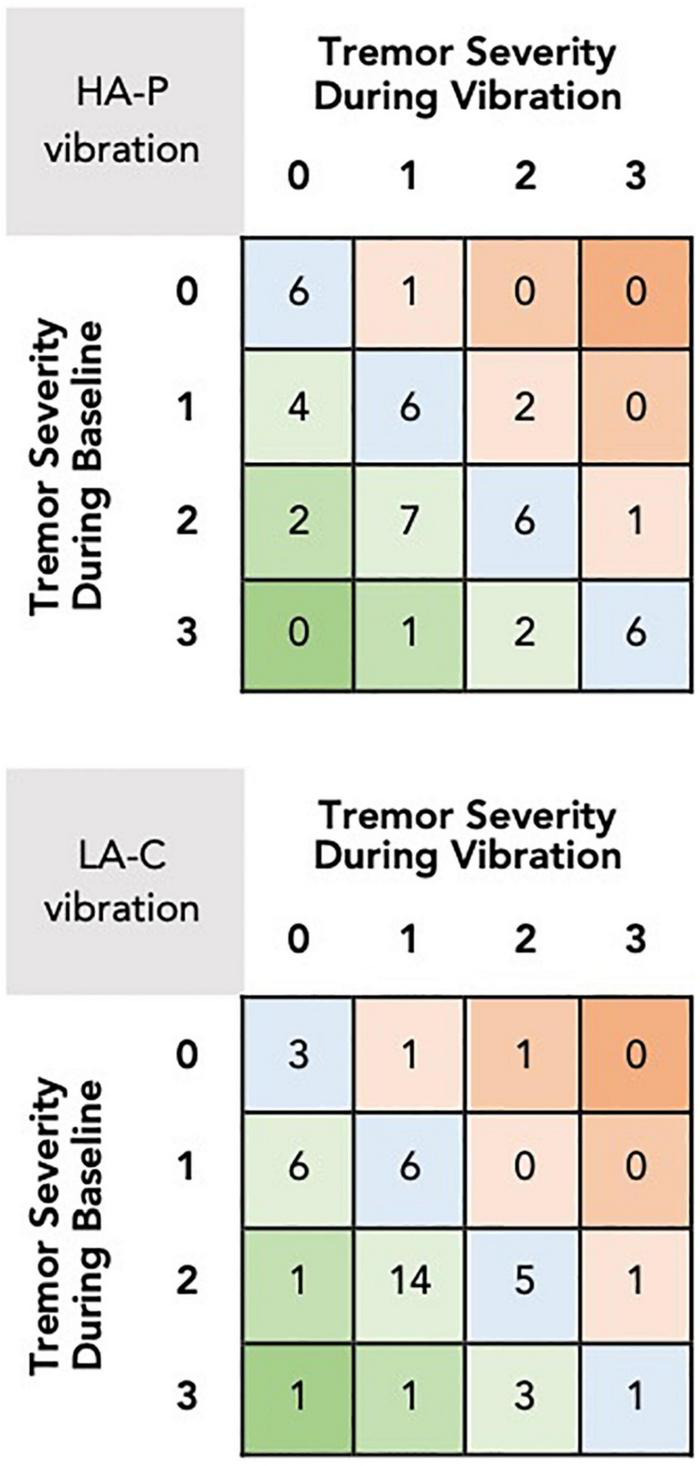
Changes in median resting tremor severity scores between baseline and during vibration for the HA-P (top) and the LA-C (bottom) vibration trials. The rows and columns of the grid refer to the tremor severity scores during baseline and vibration, respectively, with each square displaying the number of subjects who received these scores. Squares in the lower left corner of the grid represent the number of subjects that exhibited reductions in tremor severity with the vibrotactile stimulation, squares in the upper right represent increases in tremor severity, and squares in the main diagonal represent no change.

Figure 3 provides an overview of the changes in resting tremor severity score between baseline and during application of the vibrotactile stimulation. For the HA-P vibration trial, 16 subjects exhibited a decrease in median resting tremor severity compared to four subjects showing an increase, while 24 subjects exhibited no change. Similarly, for the LA-C vibration trial, 26 subjects exhibited a decrease in median tremor severity compared to three subjects exhibiting an increase, while 15 subjects displayed no change.

The multilevel models identified significant differences in tremor severity between baseline and HA-P [*t*_(88.0)_ = 3.39, *p* < 0.001, *r* = 0.54], and baseline and LA-C [*t*_(88.8)_ = 4.80, *p* < 0.001, *r* = 0.37]. No difference was identified between HA-P and LA-C with [*t*_(42.0)_ = 2.04, *p* = 0.16] or without controlling for each subject’s baseline tremor severity score [*t*_(89.5)_ = 1.50, *p* = 0.14].

## Discussion

Our results demonstrated that two different paradigms of 10 min of vibrotactile stimulation of the wrists and ankles using a novel set of wearable devices was safe and well tolerated by individuals with Parkinsonian resting tremor. The associated effect sizes were moderate in both the HA-P and LA-C vibration paradigms, with only a small number of subjects exhibiting an abolition of resting tremor (HA-P: *n* = 5; LA-C: *n* = 6) or a reduction of more than one point (HA-P: *n* = 3; LA-C: *n* = 3). These effects were not as pronounced as those frequently observed by pharmaceutical or surgical intervention (Bejjani, 2000), however, as patients often abandon Parkinson’s medications due to side effects, the demand for well-tolerated auxiliary therapies remains considerable.

The neurological mechanism by which vibrotactile stimulation may relieve motor symptomology of PD is not fully described, but may be related to the pathophysiology of PD. Dopamine depletion leads to pathologically increased neuronal synchronization in the beta frequency (15–30 Hz) band throughout the basal ganglia, thalamus and sensorimotor cortex (Brittain and Brown, 2014). Disruptions in synchronization in this frequency band are associated with improvements in motor symptoms (Kuhn et al., 2008). Tactile stimulation of the skin causes a decrease in synchronous beta band activity in the sensorimotor cortex (Gaetz and Cheyne, 2006). Therefore, it is plausible that vibratory input to the skin may be capable of disrupting the pathological beta activity observed in PD and relieving the accompanying motor symptomology (Sharififar et al., 2014; Syrkin-Nikolau et al., 2018).

The main limitation of the current study was the sample size and also the potential for a placebo effect given that both stimulation paradigms revealed clinical benefits. The inclusion of an adequate sham condition is pertinent in PD as expectations of benefit can lead to dopaminergic activation (de la Fuente-Fernández et al., 2001) and this pilot trial was important as it revealed that the two conditions were active stimulation and therefore could not be considered a sham for future trials. An additional limitation of the current study was that the duration of the safety and tolerability evaluation was quire short and therefore does not provide us with information regarding safety and tolerability of this technology in an extended home use context. Further investigation of the current wearable devices is therefore required to determine how the moderate benefits observed in the current investigation compare to placebo responses, and to evaluate safety and tolerability of the technology in a home environment.

## Conclusion

In conclusion, this pilot study demonstrated that short durations of vibrotactile stimulation delivered *via* wearable devices is a safe and feasible intervention stimulus in individuals with PD, and may confer a mild to moderate relief of resting tremor severity. Future research should examine the effects of extended home use of wearable devices on a broader range of motor impairments.

## Data Availability Statement

The raw data supporting the conclusions of this article will be made available by the authors, without undue reservation.

## Ethics Statement

The studies involving human participants were reviewed and approved by the Program for Protection of Human Subjects at Icahn School of Medicine at Mount Sinai (IRB 17-00555). The patients/participants provided their written informed consent to participate in this study.

## Author Contributions

DP conceptualized the study. AF and TP organized and executed the project. SB designed and executed statistical analysis. DP, LT, and AF prepared the first manuscript draft. All authors reviewed and approved the final version of the manuscript.

## Conflict of Interest

The authors declare that the research was conducted in the absence of any commercial or financial relationships that could be construed as a potential conflict of interest.

## Publisher’s Note

All claims expressed in this article are solely those of the authors and do not necessarily represent those of their affiliated organizations, or those of the publisher, the editors and the reviewers. Any product that may be evaluated in this article, or claim that may be made by its manufacturer, is not guaranteed or endorsed by the publisher.

## References

[B1] Bejjani B.-P. (2000). Axial parkinsonian symptoms can be improved: the role of levodopa and bilateral subthalamic stimulation. *J. Neurol. Neurosurg. Psychiatry* 68 595–600. 10.1136/jnnp.68.5.595 10766889 PMC1736917

[B2] Brittain J.-S. Brown P. (2014). Oscillations and the basal ganglia: motor control and beyond. *NeuroImage* 85 637–647. 10.1016/j.neuroimage.2013.05.084 23711535 PMC4813758

[B3] de la Fuente-Fernández R. Ruth T. J. Sossi V. Schulzer M. Calne D. B. Stoessl A. J. (2001). Expectation and dopamine release: mechanism of the placebo effect in Parkinson’s disease. *Science* 293 1164–1166. 10.1126/science.1060937 11498597

[B4] Deuschl G. Krack P. Bötzel K. Dillmann U. Gruber D. Hilker R. (2006). A randomized trial of deep-brain stimulation for Parkinson’s disease. *N. Engl. J. Med.* 13 896–908. 10.1056/NEJMoa060281 16943402

[B5] Gaetz W. Cheyne D. (2006). Localization of sensorimotor cortical rhythms induced by tactile stimulation using spatially filtered MEG. *NeuroImage* 30 899–908. 10.1016/j.neuroimage.2005.10.009 16326116

[B6] Gaßner H. Janzen A. Schwirtz A. Jansen P. (2014). Random whole body vibration over 5 weeks leads to effects similar to placebo: a controlled study in Parkinson’s disease. *Parkinsons Dis.* 2014:386495. 10.1155/2014/386495 25371843 PMC4211146

[B7] Haas C. T. Turbanski S. Kessler K. Schmidtbleicher D. (2006). The effects of random whole-body-vibration on motor symptoms in Parkinson’s disease. *NRE* 21 29–36. 10.3233/NRE-2006-21105 16720935

[B8] Hughes A. J. Daniel S. E. Blankson S. Lees A. J. (1993). A clinicopathologic study of 100 cases of Parkinson’s disease. *Arch. Neurol.* 50 140–148. 10.1001/archneur.1993.00540020018011 8431132

[B9] Kalia L. V. Lang A. E. (2015). Parkinson’s disease. *Lancet* 386 896–912. 10.1016/S0140-6736(14)61393-3 25904081

[B10] Kapur S. S. Stebbins G. T. Goetz C. G. (2012). Vibration therapy for Parkinson’s disease: charcot’s studies revisited. *J Parkinsons Dis.* 2 23–27. 10.3233/JPD-2012-12079 23939405

[B11] Kestenbaum M. Ford B. Louis E. D. (2015). Estimating the proportion of essential tremor and Parkinson’s disease patients undergoing deep brain stimulation surgery: five-year data from columbia university medical center (2009-2014). *Mov. Disord. Clin. Pract.* 2 384–387. 10.1002/mdc3.12185 28845438 PMC5571873

[B12] King L. K. Almeida Q. J. Ahonen H. (2009). Short-term effects of vibration therapy on motor impairments in Parkinson’s disease. *NRE* 25 297–306. 10.3233/NRE-2009-0528 20037223

[B13] Kowal S. L. Dall T. M. Chakrabarti R. Storm M. V. Jain A. (2013). The current and projected economic burden of Parkinson’s disease in the united states: economic burden of PD in The US. *Mov. Disord.* 28 311–318. 10.1002/mds.25292 23436720

[B14] Kuhn A. A. Kempf F. Brucke C. Gaynor Doyle L. Martinez-Torres I. Pogosyan A. (2008). High-frequency stimulation of the subthalamic nucleus suppresses oscillatory activity in patients with Parkinson’s disease in parallel with improvement in motor performance. *J. Neurosci.* 28 6165–6173. 10.1523/JNEUROSCI.0282-08.2008 18550758 PMC6670522

[B15] Louis E. D. Klatka L. A. Liu Y. Fahn S. (1997). Comparison of extrapyramidal features in 31 pathologically confirmed cases of diffuse lewy body disease and 34 pathologically confirmed cases of parkinson’s disease. *Neurology* 48:376. 10.1212/WNL.48.2.376 9040725

[B16] Morgante L. Morgante F. Moro E. Epifanio A. Girlanda P. Ragonese P. (2007). How many parkinsonian patients are suitable candidates for deep brain stimulation of subthalamic nucleus? Results of a questionnaire. *Park. Relat. Disord.* 13 528–531. 10.1016/j.parkreldis.2006.12.013 17347021

[B17] Pasquini J. Ceravolo R. Qamhawi Z. Lee J.-Y. Deuschl G. Brooks D. J. (2018). Progression of tremor in early stages of Parkinson’s disease: a clinical and neuroimaging study. *Brain* 141 811–821. 10.1093/brain/awx376 29365117

[B18] Ramirez-Zamora A. Molho E. (2014). Treatment of motor fluctuations in Parkinson’s disease: recent developments and future directions. *Exp. Rev. Neurother.* 14 93–103. 10.1586/14737175.2014.868306 24328720

[B19] Sharififar S. Coronado R. A. Romero S. Azari H. Thigpen M. (2014). The effects of whole body vibration on mobility and balance in Parkinson disease: a systematic review. *Iran J. Med. Sci.* 39 318–326. 25031483 PMC4100042

[B20] Syrkin-Nikolau J. Neuville R. O’Day J. Anidi C. Koop M. M. Martin T. (2018). Coordinated reset vibrotactile stimulation shows prolonged improvement in Parkinson’s disease. *Mov. Disord.* 33 179–180. 10.1002/mds.27223 29150859 PMC5836884

